# Comparing radiological response to radiofrequency ablation, percutaneous ethanol injection, and transarterial chemoembolization in patients with hepatocellular carcinoma on the waiting list for liver transplant

**DOI:** 10.31744/einstein_journal/2025AO1315

**Published:** 2025-11-07

**Authors:** Bruna De Fina, Felipe Nasser, Breno Boueri Affonso, Francisco Leonardo Galastri, Leonardo Guedes Moreira Valle, Marcela Juliano Silva Cunha, Guilherme Cayres Mariotti, Ary Serpa, Rodrigo Gobbo Garcia, Nelson Wolosker

**Affiliations:** 1 Hospital Israelita Albert Einstein São Paulo SP Brazil Hospital Israelita Albert Einstein, São Paulo, SP, Brazil.; 2 Hospital Israelita Albert Einstein Hospital Municipal Dr. Gilson de Cássia Marques de Carvalho São Paulo SP Brazil Hospital Municipal Dr. Gilson de Cássia Marques de Carvalho; Hospital Israelita Albert Einstein, São Paulo, SP, Brazil.

**Keywords:** Hepatocellular carcinoma, Chemoembolization therapy, Radiofrequency ablation, Catheter ablation, Liver transplantation, Liver neoplasms, Ethanol, Waiting lists

## Abstract

**Objective:**

We aimed to compare the radiological response in small hepatocellular carcinomas treated with radiofrequency ablation, percutaneous ethanol injection, and transarterial chemoembolization using the modified Response Evaluation Criteria in Solid Tumor radiological evaluation in patients on the waiting list for liver transplant.

**Methods:**

A retrospective analysis was conducted on 98 patients with uninodular tumors of 2–3cm, who were on the waiting list for liver transplantation and underwent radiofrequency ablation, percutaneous ethanol injection, or transarterial chemoembolization as bridging therapies. The patients underwent clinical and radiological follow-up and were assessed for necrosis and radiological response rates using control magnetic resonance imaging after 30–60 days.

**Results:**

Among the patients, 19, 25, and 54 were in the percutaneous ethanol injection, radiofrequency ablation, and transarterial chemoembolization groups, respectively. Most patients were male, had hepatitis C-induced liver disease, and exhibited a Child-Pugh A status. Imaging control evaluation showed that the radiofrequency ablation group had the highest necrosis rate (97.12%), followed by the percutaneous ethanol injection (77.05%) and transarterial chemoembolization (76.2%) groups (p=0.002). The radiofrequency ablation group showed the highest complete response rate (96%); the percutaneous ethanol injection and transarterial chemoembolization groups had 63.2% and 46.3%, respectively (p<0.001).

**Conclusion:**

Radiofrequency ablation resulted in a higher necrosis rate and superior radiological response than did percutaneous ethanol injection and transarterial chemoembolization in this study cohort.

## In Brief

Minimally invasive neoadjuvant treatments, such as radiofrequency ablation, percutaneous ethanol injection, and transarterial hepatic chemoembolization, are usually recommended for patients on the transplant waiting list to prevent disease progression and dropout. Radiofrequency ablation showed a higher necrosis rate and superior radiological response than did percutaneous ethanol injection and transarterial chemoembolization in this cohort.

## Highlights

■ Minimally invasive neoadjuvants are usually recommended for patients on the transplant waiting list.
■ Radiofrequency ablation showed a higher necrosis rate and superior radiological response than did percutaneous ethanol injection and transarterial chemoembolization in this study cohort.

## Graphical abstract

**Figure:**
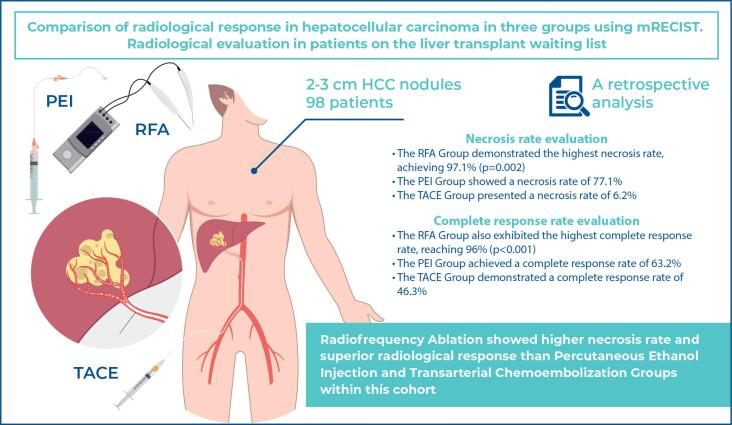

## INTRODUCTION

Hepatocellular carcinoma (HCC) is the fifth leading cause of cancer and the third leading cause of cancer-related death worldwide. It is usually associated with liver disease and cirrhosis.^(1)^

According to the clinical practice guidelines of the European Association for the Study of the Liver, patients with a single nodule of<2cm and preserved liver function may undergo curative resection or ablation. However, most patients with HCC are diagnosed with impaired liver function and multiple or larger nodules. These cases are classified according to the Milan criteria, which include the occurrence of a single hepatocellular carcinoma of 5cm or three hepatocellular carcinomas of <3cm. Patients who meet these criteria are recommended for liver transplantation, which is considered a curative therapy. Those whose conditions are inconsistent with the criteria are offered palliative treatment.^(2)^

In many countries, the demand for organ transplants exceeds the supply, leading to waiting lists of over a year. A major concern is the time patients spend on waiting lists, during which 15–30% experience disease progression. Patients whose conditions do not meet the Milan criteria are excluded from the list, as they become ineligible for curative treatment.^(2)^

Minimally invasive neoadjuvant treatments, such as radiofrequency ablation (RFA), percutaneous ethanol injection (PEI), and transarterial hepatic chemoembolization (TACE), are common recommendations for patients on the transplant waiting list to prevent disease progression and dropout.^(3)^ No single strategy has proven to be superior in terms of tumor response, dropout from the waiting list, and post-transplant outcomes.^(4–6)^

## OBJECTIVE

We aimed to compare the radiological responses of 2-3cm nodules treated with radiofrequency ablation, percutaneous ethanol injection, or transarterial hepatic chemoembolization using the modified Response Evaluation Criteria in Solid Tumor radiological evaluation in patients with hepatocellular carcinoma on the waiting list for liver transplant.

## METHODS

A retrospective analysis was conducted on patients with HCC with uninodules of 2-3cm who were on the waiting list for liver transplantation and underwent three types of bridging treatments between 2011 and 2020 at *Hospital Israelita Albert Einstein* and *Hospital Municipal Dr. Gilson de Cássia Marques de Carvalho* São Paulo, Brazil. The locoregional treatments (LRTs) included RFA, PEI, and TACE.

Demographic, procedural, and radiological response data were collected from a prospectively filled dataset from the hospital departments of interventional radiology and liver transplantation. These data were approved by the local institutional review board.

### Eligibility criteria for patient selection

The following inclusion criteria were considered: incidence of hepatocarcinoma tumor stage A according to the Barcelona Clinic Liver Cancer^(1)^ and being within the Milan criteria,^(7)^ Child-Pugh scores of A and B, and HCC cases meeting the criteria for liver transplantation. All patients were indicated for neoadjuvant treatments according to the institutional protocol of the liver transplant team after a multidisciplinary discussion, where anatomical and clinical characteristics were considered to indicate percutaneous (RFA or PEI) or TACE therapy.

The exclusion criteria were as follows: vascular invasion or extrahepatic dissemination; age <18 or >70 years; refractory or intractable ascites; Grade III/IV hepatic encephalopathy; complete thrombosis of the main trunk of the portal vein; total bilirubin levels exceeding 3mg/dL; coagulation disorders (INR>2 and/or platelets <20,000); performance score (PST)>2; moderate or severe hydrothorax; renal failure with creatinine clearance rate of <30mL/h; calculated MELD score >30; or technical impediments to perform the procedures.

### Intervention protocols

Radiofrequency ablation and PEI were performed in the angiography suite under ultrasound (US) guidance using 16-slice multidetector computed tomography (CT). All procedures were performed percutaneously by two experienced interventional radiologists. Anesthesia modalities included general anesthesia with intravenous sedation and local anesthesia. RFA was performed in a single session using an RFA 200-W generator device. Radiofrequency current was emitted for 12 min in each cycle, with CT and US performed after each cycle to assess the ablation margins (>1cm). If the scan showed incomplete ablation, multiple overlapping RFA ablations were performed to achieve complete tumor ablation. Preprocedural hydrodissection was performed if untargeted structures were at risk.

Percutaneous ethanol injectio procedures were performed in multiple sessions (up to three sessions with a 1-week interval) using a 15- or 20cm long, 22-gauge Turner needle. Absolute ethanol was injected following a contrast-enhanced CT using a 20% iodinated contrast solution to evaluate the adequate positioning of the needle. The injected volume was based on the tumor volume estimated from the preprocedural CT, assuming a 1:1 ratio (range, 4–20mL). Patients who underwent RFA were routinely admitted to the hospital following the procedure to ensure their discharge 1 day after the procedure. Patients who underwent PEI were discharged from the hospital 6 h after the procedure in the absence of complications.

Transarterial hepatic chemoembolization procedures were performed at the Image-Guided Intervention Center by the Vascular Interventional Radiology team comprising three experienced interventional radiologists. The procedures were performed under local anesthesia with lidocaine 2%, sedation, and analgesia via the venous administration of midazolam and fentanyl. Using a femoral artery approach, diagnostic superior mesenteric, celiac trunk, and common hepatic artery angiograms were performed using a Cobra 2 5F or Simmons 2 5F (Cordis, USA) to outline the hepatic artery anatomy, delineate the tumor, identify the feeding vessels of the tumor, and evaluate portal vein patency. The feeding vessels were catheterized with a 2.8 F microcatheter (Progreat, Terumo, Japan), and the tumors were embolized by injecting iodinated contrast medium mixed with one vial of DC-BEAD (Biocompatibles, UK) or HepaSphere (Merit Medical Systems, USA) impregnated with 50mg of doxorubicin, following the respective manufacturers’ instructions. The embolization endpoint was near the stasis observed in the artery feeding the tumor. Only one session was performed for all patients.

The patients underwent clinical and radiological follow-ups using control magnetic resonance imaging 30–60 days after the procedure. Tumor response to LRTs was classified as described in the mRECIST^(8,9)^ as follows:

– Complete response (CR): The disappearance of intratumoral arterial enhancement in all target lesions.
– Partial response (PR): At least a 30% decrease in the sum of the diameters of viable (contrast enhancement in the arterial phase) target lesions, taking the baseline sum of the diameters of the target lesions as the reference.
– Progressive disease (PD): An increase of at least 20% in the sum of the diameters of viable (enhancing) target lesions, taking the smallest sum of the diameters of viable (enhancing) target lesions recorded since the start of treatment as the reference.
– Stable disease (SD): Any case ineligible for PR or PD.

All patients provided informed consent for data analysis, which was approved by the Research Ethics Committees of *Hospital Israelita Albert Einstein (CAAE: 29351020.9.0000.0071, # 3.957.784) and Hospital Municipal Gilson de Cássia Marques de Carvalho (CAAE: 68277917.4.0000.0071; # 3903459)*.

### Statistical analysis

Patients were categorized into treatment groups. Quantitative variables were compared between groups using analysis of variance or Kruskal–Wallis tests, followed by Bonferroni multiple comparisons. Qualitative variables (sex, etiology, and Child-Pugh classification) were described according to group, and the association was verified using likelihood ratio tests.

## RESULTS

The demographic and clinical data of the 98 nodules in the 98 patients are summarized in table 1. Among these patients, 19, 25, and 54 were in the PEI, RFA, and TACE Groups, respectively. Most patients were male, had hepatitis C-induced liver disease, and assumed the Child-Pugh A status (Table 1). Considering the nodule size, the TACE Group had the largest mean size (2.54cm), followed by the RFA (2.29 cm) and PEI (2.17cm) groups (p=0.001).

**Table 1:** Patient's demographics

Variable		TACE Group n (%)	RFA Group n (%)	PEI Group n (%)	Total n (%)	p value
Sex						0.477
	Female	10 (18.5)	3 (12)	5 (26.3)	18 (18.4)	
	Male	44 (81.5)	22 (88)	14 (73.7)	80 (81.6)	
	Total	54 (100)	25 (100)	19 (100)	98 (100)	
Cause of liver disease						0.093
	Hepatitis B virus	3 (5.8)	2 (8.3)	0 (0)	5 (5.4)	
	Hepatitis C virus	27 (51.9)	13 (54.2)	5 (29.4)	45 (48.4)	
	Alcoholic	9 (17.3)	7 (29.2)	8 (47.1)	24 (25.8)	
	Others	13 (25)	2 (8.3)	4 (23.5)	19 (20.4)	
	Total	52 (100)	24 (100)	17 (100)	93 (100)	
CHILD-PUGH						0.140
	A	24 (48)	15 (62.5)	12 (70.6)	51 (56)	
	B	22 (44)	9 (37.5)	5 (29.4)	36 (39.6)	
	C	4 (8)	0 (0)	0 (0)	4 (4.4)	
	Total	50 (100)	24 (100)	17 (100)	91 (100)	

TACE: transarterial chemoembolization; RFA: radiofrequency ablation; PEI: percutaneous ethanol injection.

The necrosis rate evaluation showed that the RFA Group achieved the highest necrosis rate (mean, 97.12%), followed by the PEI (mean, 77.05%) and TACE (76.2%) groups (p=0.009) (Table 2).

**Table 2:** Necrosis rate

Group	Median (%)	DP (%)	n	p value
TACE	76.20	31.38	54	0.009
RFA	97.12	14.40	25	
PEI	77.05	33.21	19	
Total	81.70	29.60	98	

TACE: transarterial chemoembolization; RFA: radiofrequency ablation; PEI: percutaneous ethanol injection.

For the response rates among the three treatment groups, the RFA Group exhibited the highest CR rate (96%, p=0.001), with no patients showing PR, and only one presenting with SD. The TACE Group demonstrated a CR rate of 46.3%, with 42.6% of the patients exhibiting PR, and 11.1% showing SD. In the PEI Group, 63.2% of patients achieved CR, 26.3% achieved PR, and 9.2% exhibited SD (Table 3). No patient exhibited PD.

**Table 3:** Comparison of response

	TACE Group n (%)	RFA Group n (%)	PEI Group n (%)	Total n (%)	p value
					0.001
SD	6 (11.1)	1 (4.0)	2 (10.5)	9 (9.2)	
PR	23 (42.6)	0 (0.0)	5 (26.3)	28 (28.6)	
CR	25 (46.3)	24 (96.0)	12 (63.2)	61 (62.2)	
Total	54 (100)	25 (100)	19 (100)	98 (100)	

TACE: transarterial chemoembolization; RFA: radiofrequency ablation; PEI: percutaneous ethanol injection; SD: stable disease; PR: partial response; CR: complete response.

## DISCUSSION

We compared the most frequently performed LRTs as bridge therapies among patients within the Milan criteria awaiting liver transplantation. The goal was to facilitate the determination of the optimal therapy in a given scenario, as it depends on multiple factors.

The initial discussion involved deliberating on the recommendation of locoregional bridge therapy for all patients with HCC. Clinical evidence shows that waiting list periods exceeding 6 months result in high dropout rates, owing to tumor growth; 22% of patients with HCC drop off the liver transplant waiting list, and half of these cases are owing to tumor progression.^(10–13)^ Additionally, bridge treatments are associated with improved recurrence-free and overall survival after liver transplantation, and these benefits are related to complete pathological responses. As the imaging techniques for the accurate prediction of the complete pathological response post-transplantation are lacking, sustained radiological response may be considered the most suitable surrogate endpoint in a pre-transplantation setting.^(14,15)^

No studies have been specifically designed to compare the radiological responses of TACE, RFA, and PEI as standalone therapies. In a retrospective analysis of 150 patients with HCC, Györi et al.^(5)^ compared monotherapies with combination treatments. They demonstrated that RFA exhibited the highest overall response rate (RFA [84.9%] *versus* TACE [72.0%] *versus* PEI [64.9%]), consistent with our results (RFA [97.12%] *versus* TACE [76.2%] *versus* PEI [77.05%]). Regarding CR rates, TACE, PEI, and RFA achieved 41.2%, 30.0%, and 33.3%, respectively, compared with 46.3%, 63.2%, and 96.0% in our study. In our analysis, the CR rate for RFA was higher; however, our findings are more consistent with the general literature, where RFA demonstrates CR rates exceeding 90% and superior radiological responses compared with those of other LRTs such as TACE and PEI.^(16)^

In our study, the TACE Group had the lowest CR rate (less than half of the nodules achieved complete necrosis) and the highest proportion of patients with PR (42.6%). However, PR was less common in the PEI Group (26.3%) and was not observed in the RFA Group. This variation in the distribution of TACE results, as also reported in other studies, may have been influenced by the presence of larger tumors in this group and by the limited one-session procedure.^(17–20)^

Not all treatment modalities are covered by the Brazilian public healthcare system (SUS) and are, therefore, unavailable to all patients. This highlights the significance of evaluating different LRTs and how they may enhance HCC pre- and post-liver transplant outcomes.

Cost analyses and public policy decisions have significantly influenced the LRT choice. In Brazil, the cost of a PEI session is less than USD 200 per session. The TACE procedure costs approximately USD 2,000, and RFA costs approximately USD 3,000. Although cost-effectiveness studies have demonstrated the superiority of RFA over PEI, even considering the higher isolated cost of the former, the Brazilian transplant program covers PEI and TACE procedures, but not RFA. This limitation reduces the indications for RFA as a curative treatment or bridge to transplantation.^(21,22)^

Radiofrequency ablation is the most common technique for <3-cm nodules, and in most studies, it has demonstrated the highest necrosis and CR rates, as found in our study. Despite its many potential favorable effects, this technique has limitations such as restricted ablation volume, anatomical technical constraints, complications depending on tumor location, heat-sink effect, intravascular tumor spreading owing to intratumoral pressure during RFA, and tumor seeding.^(23–26)^

Percutaneous ethanol injection involves the direct injection of absolute ethanol into targeted tumors using fine needles under ultrasonographic guidance. It is a cost-effective, well-tolerated, and simple-to-perform ablation technique. Hence, PEI usually presents the lowest and most variable rates of necrosis response and CR. In a recent meta-analysis, Yang et al. found variability in the CR rates among patients undergoing PEI, ranging from 100% to 65.6%.^(27–29)^

Transarterial hepatic chemoembolization is applicable across different sizes and numbers of nodules, suitable at various disease stages, and useful for other purposes (adjuvant, bridging, downstaging, and palliative treatments), making it a common technique in specialized services for these patients. Transarterial hepatic chemoembolization showed the optimum results for large nodules and complex anatomies with a high complication risk.^(30–32)^

Studies designed to compare dropout rates and post-liver transplantation survival following the application of LRT as a bridging treatment have not shown significant differences between groups. A Brazilian study reported dropout rates below 8.9% after PEI and 14% after TACE, with similar 5-year post-transplant recurrence-free survival rates. These findings support the use of the PEI and TACE techniques in this setting and encourage the use of combined techniques to improve outcomes.^(5,33,34)^

Our study had some limitations. Understanding the response rate is essential for identifying the optimal technique; however, the lack of follow-up limits the correlation between these results and clinical response. Determining the dropout and survival rates for each procedure after liver transplantation poses a complex challenge and should be focused on in future investigations. Additionally, variables such as the molecular biology of each nodule and its specific characteristics are crucial factors, necessitating their consideration in these analyses.

## CONCLUSION

Radiofrequency ablation showed the highest necrosis rate and a complete radiological response. Factors such as the cost and availability should be considered in the decision-making process.
